# Rapid and efficient one-step generation of paired gRNA CRISPR-*Cas9* libraries

**DOI:** 10.1038/ncomms9083

**Published:** 2015-08-17

**Authors:** Joana A. Vidigal, Andrea Ventura

**Affiliations:** 1 Memorial Sloan Kettering Cancer Center, Cancer Biology and Genetics Program, 1275 York Avenue, New York, New York 10065, USA

## Abstract

The CRISPR-*Cas9* system is a powerful tool to edit eukaryotic genomes that has recently been adapted for functional screens. Several of its applications—including the disruption of genes using *Cas9*-nickase and the generation of large deletions—require co-expression of two distinct guide RNAs (gRNAs). However, the lack of experimental approaches to generate pools of paired gRNA vectors prevents these applications from being scalable. Here we report a simple, inexpensive, one-step method that allows for the rapid and efficient cloning of gRNA pairs into expression vectors. We show that this method can be used to generate pooled libraries and is therefore suitable for *in vivo* and *in vitro* functional screens.

## Abstract (web summary)

CRISPR-Cas9 is a powerful tool for genome editing; however, difficulties in generating pools of paired guide RNAs limit its applicability to large-scale screening experiments. Here the authors report a one-step method for rapid and efficient generation of pooled libraries of guide RNA pairs.

## Graphical abstract

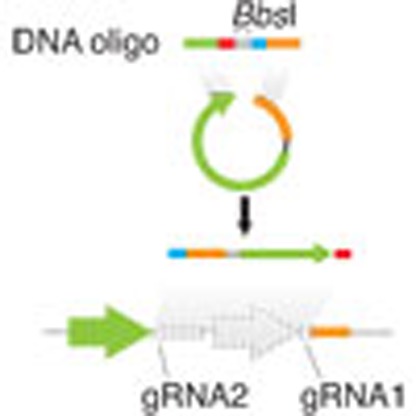

The CRISPR-*Cas9* system for genome editing is a powerful tool for functional screens *in vitro* and *in vivo*^1^,^2^. Co-expression of the bacterial *Cas9* endonuclease and a short guide RNA molecule (gRNA) is sufficient to generate double-stranded DNA breaks in eukaryotic cells. These cuts occur at sites that have a short (∼20 nucleotides) homology to the 5’ end of the gRNA and are followed by an NGG sequence—known as the protospacer-adjacent motif^1^,^3^.

In eukaryotes, double-stranded DNA breaks are primarily repaired through the error-prone non-homologous end-joining pathway^4^, often leading to the generation of small indels at the target site. Thus, the CRISPR-*Cas9* system provides a simple way of disrupting the frame of protein-coding genes to produce loss of function alleles^2^,^5^,^6^,^7^,^8^,^9^,^10^. Gene disruption using the CRIPSR-*Cas9* system has proven highly efficient and has been used for biallelic targeting of multiple genes simultaneously^6^,^11^,^12^,^13^. Because target recognition by *Cas9* requires only a short homology between the gRNA and the genomic locus, the CRISPR-*Cas9* system is suitable for large scale loss of function screens. Libraries of vectors expressing gRNAs designed to target genes of interest can be easily and inexpensively generated in one cloning step starting from on-chip synthesized oligonucleotides pools^8^,^9^,^10^, a strategy that has been successfully applied to the generation of RNAi libraries^14^.

However, a growing number of applications of the CRISPR-*Cas9* system require the simultaneous expression of two guide RNAs transcribed from independent promoters. For instance, a strategy that significantly reduces off-target mutations while retaining on-target cutting efficiency relies on the expression of a mutant Cas9 having one of the two nuclease domains disrupted (Cas9n; nickase) together with two gRNAs targeting off-set sites on opposite DNA strands^15^,^16^.

Paired gRNAs can also be used to produce genomic deletions^13^,^17^,^18^, which (among other uses) extends the application of CRISPR-based knockout studies to the noncoding portion of the genome^19^.

Because the sequences encoding the gRNA pairs need to be cloned downstream of independent promoters in the same plasmid, available strategies to rapidly generate pooled libraries^14^ cannot be used. This severely limits the scalability of this approach for functional screens. To overcome this limitation, we have developed a simple one-step method that allows the rapid and efficient cloning of specific gRNA-pairs into virtually any CRISPR-expression vector starting from pools of short oligonucleotides. This methods uses an intermediate circularization-linearization step that ensures that the two gRNAs are cloned downstream of independent U6 promoters in the final plasmid. Importantly, because the sequence of the two gRNAs paired in the dual-expression construct is determined at the oligonucleotide design step, correct pairing of gRNAs for the same genomic locus in the resulting plasmid is ensured by default in our experimental scheme.

We also show that lentiviral vectors expressing gRNA pairs from two identical U6 promoters are prone to recombination and consequent loss of the proximal gRNA. We overcome this problem by generating a novel lentiviral vector containing a human U6 promoter and a modified murine U6 promoter in which key regulatory sequences are replaced by the human equivalents.

By providing a simple, fast and inexpensive way to generate pooled library of paired gRNAs expressing vectors this method greatly expands the potential applications of the CRISPR technology for functional genomic studies.

## Results

### A single vector for co-delivery of *Cas9* and paired gRNAs

To determine whether a pair of gRNAs can be effectively expressed from a single vector, we tested the editing efficiency of a *Cas9* expression plasmid containing two tandem U6 promoters, each followed by unique cloning sites and a gRNA-scaffold sequence^18^.

**Figure 1:**
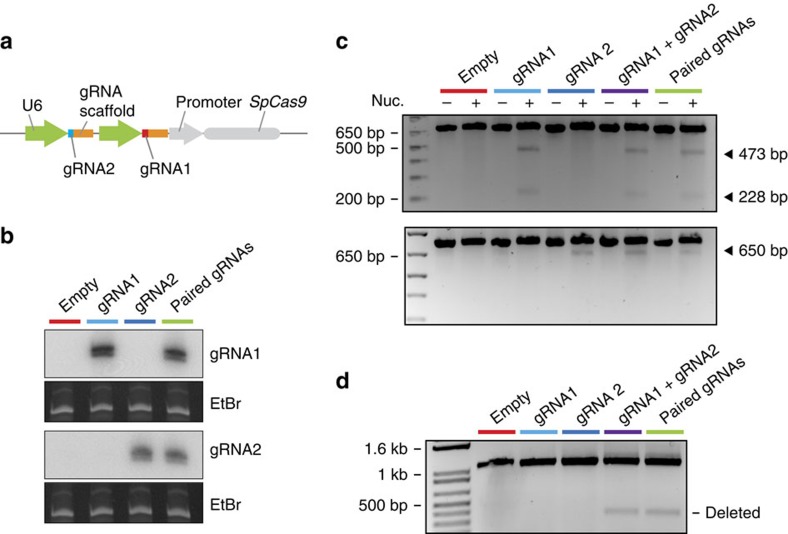
A vector for paired-gRNA/Cas9 expression. (**a**) Schematic representation of a *Cas9* expression vector containing a guide RNA pair. (**b**) Northern blot analysis to total RNA from cells transiently transfected with an empty vector (lane 1), vectors expressing single gRNAs (lanes 2 and 3) or a vector expressing an gRNA pair (lane 4). (**c**) SURVEYOR assay showing indel formation in cells transiently transfected with vectors expressing single gRNAs (lanes 3–6), an equimolar mix of the single gRNA vectors (lanes 7 and 8), or a single vector expressing the gRNA pair (lanes 9 and 10). Nuc., nuclease. (**d**) PCR analysis to targeted locus showing a ∼350 bp band corresponding the genomic deletion only in cells transfected with both gRNAs.

We designed two guide RNAs targeting two sites 760 bp apart on the murine *Trp53* locus and sequentially cloned them downstream of the two U6 promoters (Fig. 1a). Northern blot analysis showed that this plasmid configuration leads to expression of the two guides at approximately equimolar ratios (Fig. 1b). Importantly, gRNAs expressed from a single vector were able to generate on-target indels at approximately the same frequency as gRNAs co-expressed from two different plasmids (Fig. 1c) and could generate the desired genomic deletion (Fig. 1d and Supplementary Fig. 1a).

These data, together with previous work from our lab showing that tandem gRNA expression from a single vector can be used to engineer chromosomal rearrangements *in vitro* and *in vivo*^18^, demonstrate that a single plasmid expressing *Cas9* and two guide RNAs can be used for efficient genome editing.

### A one-step cloning method for paired-gRNA vectors

**Figure 2:**
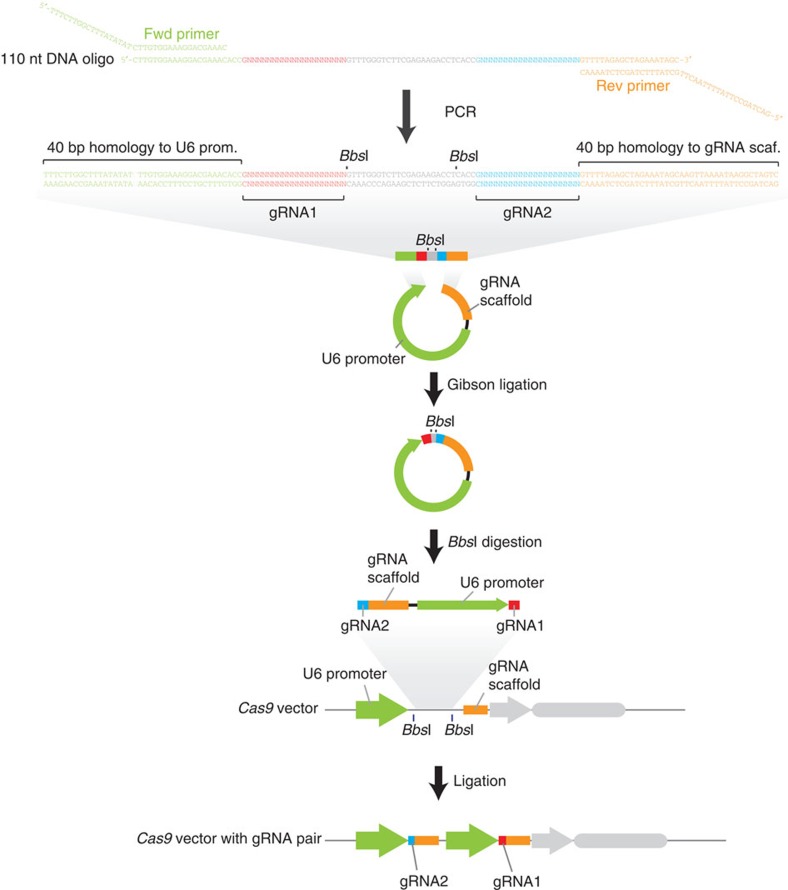
A one-step method to clone gRNAs pairs. Overview of the paired-gRNA cloning strategy. Briefly, a 110-nt DNA oligo containing the sequences of two gRNAs (represented in red and blue) is amplified by PCR, to generate a double-stranded DNA molecule that contains two restriction sites for *Bbs*I as well as 40 bp homologies to the U6 promoter and the gRNA scaffold. A Gibson reaction between the amplicon and a fragment containing a U6 promoter and an gRNA scaffold generates an intermediate circular molecule. This is linearized with *Bbs*I digestion and cloned into a *Cas9* expression plasmid to generate the final construct expressing the gRNA pair.

For the experiments described above, the two gRNA sequences were cloned sequentially in the recipient vector, a strategy that is incompatible with the generation of medium or large-scale pooled libraries. We therefore devised a method to simultaneously clone two guide RNAs starting from a short-(110 nt) DNA oligonucleotide (Fig. 2). The oligonucleotide contains the sequences corresponding to the two gRNAs separated by a short spacer harbouring two *Bbs*I sites. At the 5’ and 3’ ends of the oligo are short sequences with homology to the U6 promoter and the gRNA scaffold, respectively. Amplification of the oligo by PCR using primers that bind to these regions generates a 148-bp dsDNA molecule that contains 40 bp homologies to the 3’ end of the U6 promoter and to the 5’ end of the gRNA scaffold (Fig. 2).

In addition, we generated a plasmid (pDonor) whose digestion with *Bbs*I yields a 415 bp fragment consisting of a gRNA scaffold and a U6 promoter (Fig. 2, Supplementary Fig. 2). The presence of overlapping sequences at both ends of the Donor fragment and of the PCR product allows for their assembly into an intermediate circular molecule using the Gibson reaction^20^. Digestion of this circular intermediate with *Bbs*I produces a linear fragment with two distinct 5’ overhangs and allows for directional cloning into a variety of CRISPR-expression vectors^9^,^13^,^21^,^22^. This generates a final plasmid in which the two gRNAs present in the oligonucleotide are cloned downstream of separate U6 promoters (Figs 1a, 2).

### Pooled cloning of gRNA pairs

**Figure 3:**
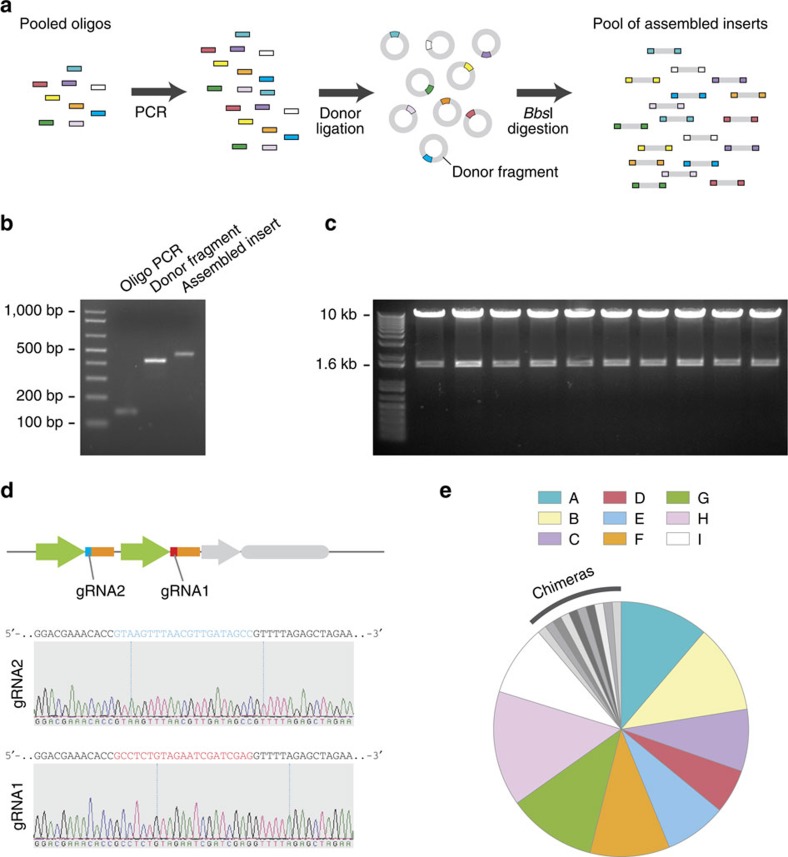
Pooled cloning of gRNA pairs. (**a**) Schematic representation of the pooled cloning strategy. Each colour represents a distinct gRNA pair. (**b**) Gel electrophoresis of the fragments used in the Gibson ligation and of the assembled insert after linearization with *Bbs*I. (**c**) *Not*I/*EcoR*I digestion of plasmid DNA showing correct vector assembly in 10/10 bacterial clones. (**d**) Example of chromatogram showing correct cloning of *oligo I* into a lentiviral vector. (**e**) Pie chart showing representation of each gRNA pair in sequenced clones.

To test whether this strategy was suitable for cloning pools of gRNA pairs, we designed oligos corresponding to nine different gRNA pairs (A-I; see Table 1) and pooled them at equimolar concentrations (Fig. 3a). We amplified the pool in a single-PCR reaction and ligated the resulting product to the U6:gRNA-scaffold fragment using the Gibson method^20^ (Fig. 3a). Linearization of the Gibson product with *Bbs*I resulted in the expected 461 bp band (Fig. 3b), which was gel purified and cloned into a linearized vector^9^ containing a U6 promoter and a gRNA scaffold (Fig. 2). Digestion of DNA from individual bacterial colonies released the expected 1.6-kb band in 10/10 clones (Fig. 3c). To determine the frequency at which individual oligos were correctly cloned, we sequenced plasmid DNA from 90 bacterial colonies. Although 10% of clones contained chimeric inserts—probably generated at the Gibson step (data not shown)—each of the remaining clones contained one of the nine gRNA pairs correctly assembled (Fig. 3d). Importantly, each pair was represented at an approximately equal frequency (min=5, max=13 and average=8.8; Fig. 3e). These results show that the method described here can be used for pooled cloning of multiple gRNA pairs.

### Paired gRNA lentiviral vectors for stable transduction of cells

CRISPR-*Cas9*-based screens have mainly relied on the stable transduction of mammalian cells with pools of lentiviral vectors^8^,^9^,^10^. Since lentiviral vectors harbouring direct repeats are unstable, the presence of two identical human U6 (hU6) promoters in the same vector might lead to viral recombination, loss of gRNA expression and consequently lower genome editing efficiency.

**Figure 4:**
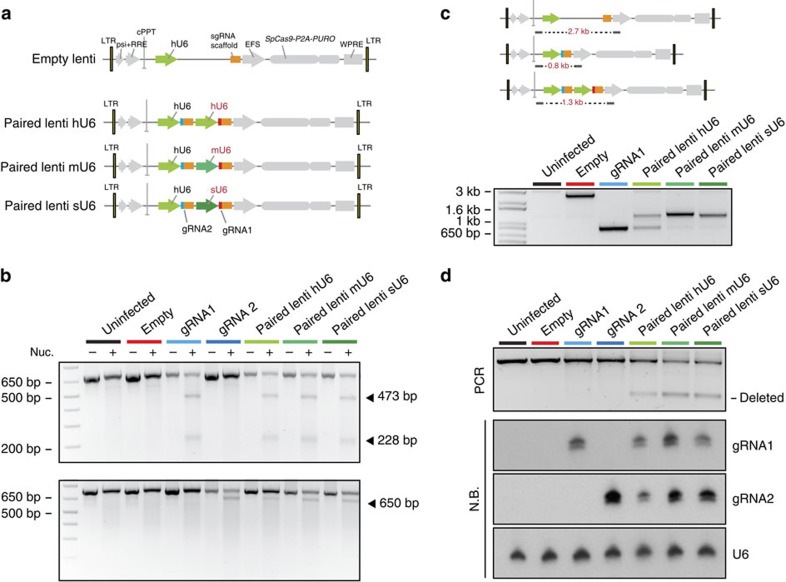
Lentiviral vectors for paired gRNA expression. (**a**) Schematic representation of the lentiviral vectors. (**b**) Detection of indel formation by SURVEYOR assay. Nuc., nuclease. (**c**) Analysis of proviral integrity by PCR to genomic DNA of infected cells. Diagram shows location of primers and expected size upon amplification of each provirus. Black bars indicate primer location. Bottom panel shows PCR assay to uninfected cells (lane 1) and cells infected with either an empty lentiviral vector (lane 2), a lentiviral vector carrying a single gRNA (lane 3), or one of the three lentiviral vectors carrying the gRNA pair (lanes 4–6). (**d**) Top, PCR analysis to targeted locus showing a band corresponding the genomic deletion only in cells infected with paired-lentiviral vectors; Bottom, northern blot (N.B.) analysis to total RNA from uninfected cells, or cells infected with the indicated lentiviral constructs.

Consistent with this hypothesis, we observed reduced editing efficiency at the 3’ cut site (targeted by the proximal gRNA; gRNA2) in cells transduced with a lentivirus expressing the two gRNAs from identical hU6 promoters (Fig. 4b; lanes 9 and 10). Genomic PCR analysis confirmed that this was due to loss of the proximal hU6-gRNA2 sequence in the proviral genome (Fig. 4c and Supplementary Fig. 1b).

We reasoned that reducing the sequence identity between the two promoters should prevent recombination. We therefore generated two additional pDonor vectors carrying the murine U6 (mU6) promoter, or a synthetic mouse U6 (sU6) promoter harbouring regulatory sequence elements from hU6 (Supplementary Fig. 2). We then used these pDonor vectors to clone the gRNA pair into a recipient lentivirus containing the hU6 promoter, thus producing two new lentiviral constructs (Fig. 4a).

In contrast to cells infected with lentiviruses carrying two hU6 promoters, cells infected with lentiviruses expressing gRNAs from two different promoters displayed largely intact proviruses (Fig. 4c). Accordingly editing efficiency at the two cut sites was comparable to what observed when each gRNA was individually expressed (Fig. 4b), as were the expression levels of the gRNAs (Fig. 4d). Finally, the desired genomic deletion was readily detectable in these cells (Fig. 4d).

These results demonstrate that the use of two different promoters prevents lentiviral recombination and allows simultaneous editing at two sites. The series of pDonor plasmids we have generated can therefore be used to rapidly build paired gRNA libraries in a variety of currently available vectors.

## Discussion

We describe here a novel method that allows the generation of pooled libraries of vectors containing paired gRNAs. The small size of the oligos that contain the gRNA sequences makes them compatible with ‘on-chip’ oligonucleotide synthesis^23^, enabling the generation of large paired gRNA pooled libraries in a fast and cost-effective manner that is analogous to what has been previously described for shRNA^14^ and single gRNA screens^8^,^9^,^10^.

In addition to providing a simple method to generate *Cas9* nickase-based^12^,^13^ pooled libraries, the method described here extends the use of CRISPR-*Cas9* screenings to a wider range of biological questions. It allows for instance the generation of deletion libraries against long-noncoding RNA genes, whose functions are unlikely to be compromised by the short insertions/deletions introduced by conventional single-guide CRISPR libraries. It also allows the generation of pooled libraries to delete chromosomal regions that are recurrently lost in human cancers, as well as the systematic functional characterization of DNA-regulatory elements.

## Methods

### DNA constructs

pDonor plasmids were generated by cloning the Donor fragments into either an *EcoR*V-digested pBluescript KS+ vector (pDonor_hU6) or into the Topo Blunt II plasmid (Invitrogene). The sU6 promoter was generated by replacing the regulatory elements of mU6 for those of hU6 (that is, the Octamer motif, the Proximal sequence element and the TATA-box). See Supplementary Fig. 2a for a schematic representation of these plasmids. The sequences contained in each of the pDonors are provided in Supplementary Fig. 2, along with the sequence of the DNA oligos used for cloning gRNA pairs under the various promoters.

### Paired gRNA cloning

For the pooled cloning oligonucleotides were mixed at equimolar concentrations and amplified with phusion polymerase (New England Biolabs) using primers that add homology regions to the 3’ region of the hU6 promoter (primer F1) and to the 5’ region of the gRNA scaffold (primer R1). The gel-purified 148-bp amplicon was ligated to the 415-bp Donor fragment—generated by *Bbs*I digestion of the pDonor plasmid—in a 3:1 molar ratio, using the Gibson Assembly Master Mix (New England Biolabs; 1 h at 50 °C). The Gibson reaction was treated with Plasmid Safe exonuclease (Epicenter) for 1 h at 37 °C to remove unligated fragments, column purified (QIAquick PCR purification kit; Qiagen), and digested with *Bbs*I at 37 °C for 3 h. The linearized 461 bp fragment was gel purified and cloned into *BsmB*I-digested lentiCRISPR vector^4^ (Addgene plasmid 49535). For 10 bacterial clones, correct assembly was confirmed by digestion of plasmid DNA with *Not*I and *EcoR*I enzymes. Sequencing of vectors was done using the F2/R2 primer set. Cloning of the paired lentivirus vectors carrying distinct pol III promoters was done as described above, but we used modified oligos and primers (Supplementary Fig. 2b and Supplementary Table 1). A list of all primer sequences and primer pairs (including corresponding amplicon sizes) used throughout this study is provided in Supplementary Tables 1 and 2, respectively. A detailed step-by-step protocol for the generation of paired gRNA libraries is provided as a Supplementary Method.

### Cell culture

Cells were cultured at 37 °C (5% CO_2_) in DME-HG supplemented with 10% FCS, L-glutamine (2 mM), penicillin (100 U ml^−1^) and streptomycin (100 μg ml^−1^). One day after seeding (1 × 10^6^ cells per well; 6-well plate), cells were transfected with 4 μg of plasmid DNA using Lipofectamine 2000 (Invitrogen) according to manufacturer’s instructions. NIH3T3 cells (ATCC; #CRL-1658) were collected either 48 h or 5 days after transfection to detect the expression of the gRNAs or the generation of genomic editing, respectively. For cell infection, 293T cells (ATCC; # CRL-3216) were transfected with lentiviral constructs together with ecotropic packaging plasmids using the protocol described above. Media containing viruses were collected 48 h after transfection and used to infect NIH3T3 cells. Infected cells were selected with puromycin (2 μg ml^−1^) for 3 days and then collected for further analysis. Analysis of proviral integrity was done by PCR using primers F2 and R2 (Supplementary Fig. 3b). In cells displaying detectable levels of proviral recombination the amplicon corresponding to the recombined viral genome was cloned into Topo Blunt II (Invitrogene) and six clones of the resulting bacterial clones sequenced.

### Northern blot analysis and detection of genomic editing

To detect gRNA expression, transduced cells were collected in TRIZOL (invitrogen) and total RNA was isolated according to manufacturers’ protocols. For each sample, 10 μg of RNA were resolved in a 15% Urea-PAGE gel and blotted onto a Hybond-N^+^ nylon membrane (GE Healthcare). Membranes were ultraviolet-cross-linked and hybridized overnight with ^32^P-labelled probes against the 5’ region of each gRNA (gRNA1 probe, TTGGACGCCCTCGCAGTGGC; gRNA2 probe, CCTGTTCGGCACACCTGCTG) and against mU6 as a loading control (GCAGGGGCCATGCTAATCTTCTCTGTATCG).

For detection of genomic deletions and indels, cells were collected in lysis buffer (100 mM Tris-HCl pH8.5, 200 mM NaCl, 5 mM EDTA, 0.2% SDS and 100 ng ml^−1^ proteinase K) and incubated at 55 °C for 4 h. Genomic DNA was extracted with phenol-chloroform followed by ethanol precipitation and amplified by PCR with Phusion polymerase (New England Biolabs). Detection of the genomic deletion was done using primers that flank the gRNA target sites (F3/R3), which leads to the amplification of a ∼1-kb band in cells carrying a wild-type locus, and a 340 bp band in cells carrying the deletion (Supplementary Fig. 3a). Sequencing of the genomic deletion was done after cloning the corresponding amplicon into Topo Blunt II vector (Invitrogene). For the detection of indel formation DNA was amplified with primers that flank the cut sites (5’cut with primers F3/R4; 3’ cut with primers F4/R3) followed by generation of DNA heteroduplexes and DNA digestion with the mismatch-sensitive SURVEYOR nuclease (Transgenomic) according to manufacturer’s instructions. Digestion fragments were resolved on a 2.5% agarose gel (Supplementary Fig. 3c,d).

## Additional information

**How to cite this article:** Vidigal, J. A. & Ventura, A. Rapid and efficient one-step generation of paired gRNA CRISPR-*Cas9* libraries. *Nat. Commun.* 6:8083 doi: 10.1038/ncomms9083 (2015).

## Supplementary Material

Supplementary Information Supplementary Figures 1-3 Supplementary Tables 1-2 and Supplementary Methods

## Figures and Tables

**Table 1:** Guide RNA sequences encoded by the DNA oligos used in the pooled clonings.

**Oligo**	**Sequence of gRNA1**	**Sequence of gRNA2**
A	5′-GCGACAAAACCGAAAATCTGT-3′	5′-GATGTCTTTAATCTACCTCGA-3′
B	5′-GCACATGTCAGCGTTTTCAAT-3′	5′-GTTCCAAGTAGGTAGATGCGA-3′
C	5′-GTTCAGTCCCAATCGAGTGC-3′	5′-GCTGGTCGCACCGAGATCTAG-3′
D	5′-GGAGAGGGGAACGGTATCCT-3′	5′-GTGACAACATATTCGGTAGTA-3′
E	5′-GAGATGACAGGGGCCATGGAG-3′	5′-GCCTTCTCAAAAAACTTACCA-3′
F	5′-GGCTATCAACGTTAAACTTA-3′	5′-GCTTCTGCCTCCGCGCAAAT-3′
G	5′-GCCCCGAGCCATGGCCGCGTC-3′	5′-GGGAAAAGTCTCCACCGGACG-3′
H	5′-GCTGGAGCTTGGGGACCTTAG-3′	5′-GTGTCCTCCCTCACACCGCTA-3′
I	5′-GCCTCTGTAGAATCGATCGAG-3′	5′-GTAAGTTTAACGTTGATAGCC-3′

gRNA, guide RNA.
