# Supplementary material for: Rapid and efficient one-step generation of paired gRNA CRISPR-Cas9 libraries
Source: Nat Commun. 2015 Aug 17;6:8083. doi: 10.1038/ncomms9083 (PMC4544769; doi:10.1038/ncomms9083)
Supplement: Supplementary Information — Supplementary Figures 1-3 Supplementary Tables 1-2 and Supplementary Methods [file ncomms9083-s1.pdf]

Supplementary Information

Vidigal and Ventura

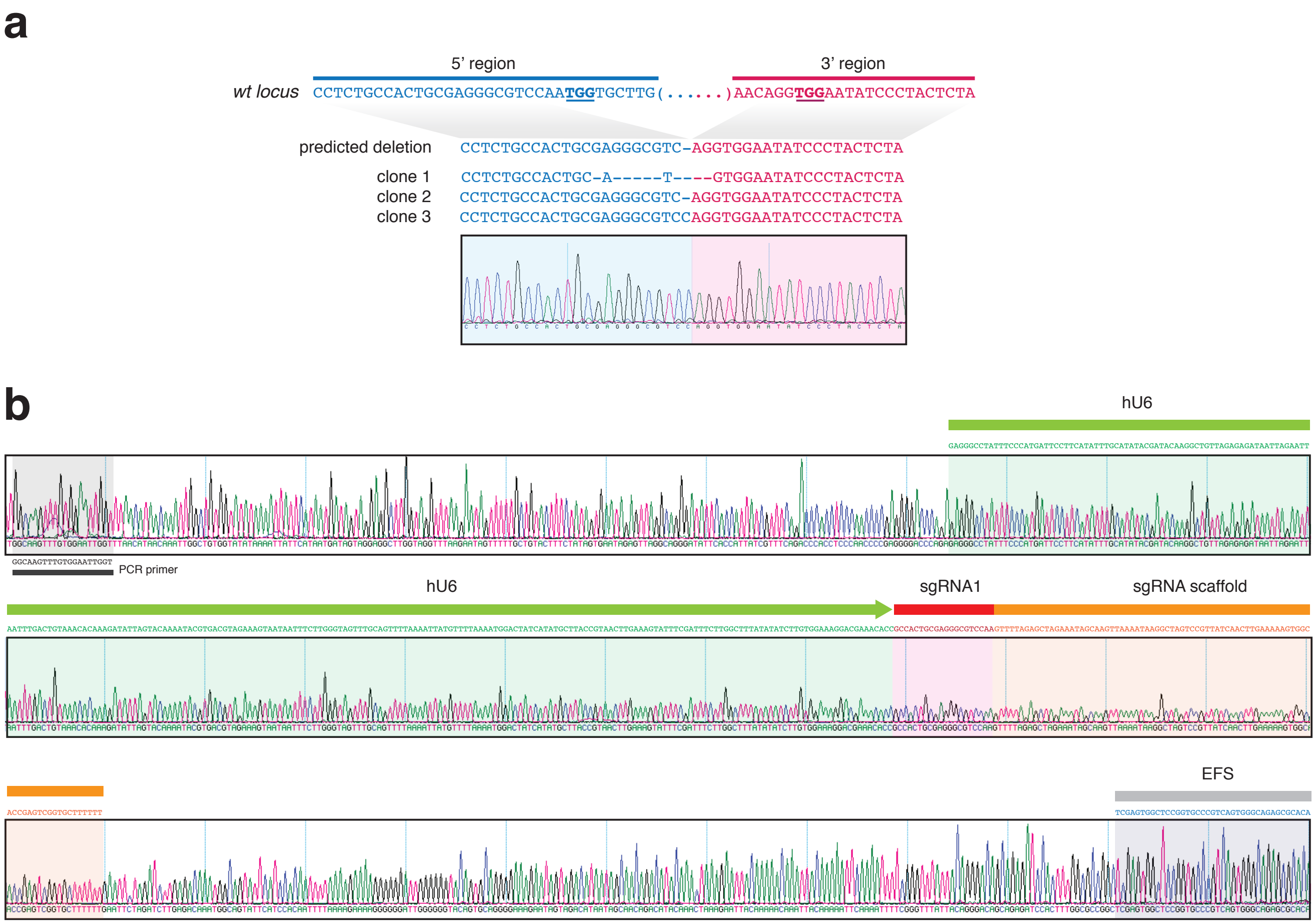

Supplementary Figure 1. Validation of PCRs to detect the genomic deletion and the integrity of the proviruses

(a) Sequencing of the genomic deletion. A schematic representation of the targeted locus as well as the predicted sequence resulting from the deletion is shown on top, with the PAM sequences of each gRNA highlighted. Below, the sequences obtained from three independent clones are shown, together with the trace data for clone 3. **(b)** Sequencing of proviral recombinants. Shown is a representative trace data of the proviral sequence of paired lenti hU6 after recombination. In all sequenced cases (6/6), recombination resulted in loss of the viral sequences flanked by the two hU6 promoters as well as loss of one hU6 sequence. Positions of the forward primer used to amplify the proviral amplicon and of relevant elements of the lentiviral vector are highlighted.

a

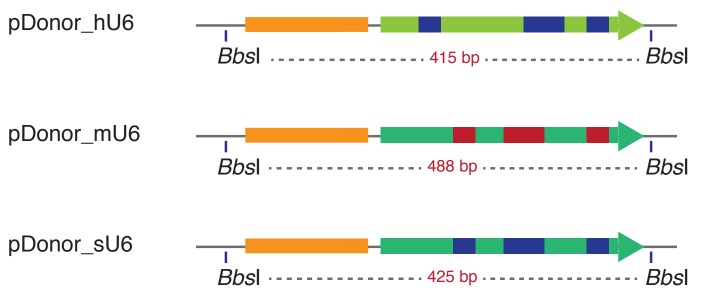

b

pDonor_hU6

GAAGACCTGTTTTAGAGCTAGAAATAGCAAGTTAAAATAAGGCTAGTCCGTTATCAACTTGAAAAAGTGGCACCGAGTCGGTGCTTTTTT
GTTTTAGAGCTAGAAATAGCAAGTTAAAATAAGGCTAGTCCGTTTTTAGCGCGTGCGCCAATTCTGCAGACAAATGGCTCTAGAGAGGGC
CTATTTCCCATGATTCCTTCAT**ATTTGCAT**ATACGATACAAGGCTGTTAGAGAGATAATTGGAATTAATTTGACTGTAAACACAAAGATA
TTAGTACAAAATACGTGACGTAGAAAGTAATAATTTCTTGGGTAGTTTGCAGTTTTAAAATTATGTTTTAAAATGGACTATCATATG**CTT**
ACCGTAACTTGAAAGTATTTCGATTTCTTGGC**TTTATATAT**CTTGTGGAAAGGACGAAACACCGTTTGGGTCTTC

pDonor_mU6

GAAGACCTGTTTTAGAGCTAGAAATAGCAAGTTAAAATAAGGCTAGTCCGTTATCAACTTGAAAAAGTGGCACCGAGTCGGTGCTTTTTT
GTTTTAGAGCTAGAAATAGCAAGTTAAAATAAGGCTAGTCCGTTTTTAGCGCGTGCGCCAATTCTGCAGACAAATGGCTCTAGATCTAGA
GATCCGACGCCGCCATCTCTAGGCCCGCGCCGGCCCCCTCGCACAGACTTGTGGGAGAAGCTCGGCTACTCCCCTGCCCCGGTTA**ATTTG**
CATATAATATTTCCTAGTAACTATAGAGGCTTAATGTGCGATAAAAGACAGATAATCTGTTCTTTTTAATACTAGCTACATTTTACATGA
TAGGCTTGGATTTCTATAAGAGATACAAATACTAAATTATTATTTTAAAAAACAGCACAAAAGGAAA**CTCACCCTAACTGTAAAGTA**ATT
GTGTGTTTTGAGAC**TATAAATAT**GCATGCGAGAAAAGCCTTGTTTGGTTTGGGTCTTC

pDonor_sU6

GAAGACCTGTTTTAGAGCTAGAAATAGCAAGTTAAAATAAGGCTAGTCCGTTATCAACTTGAAAAAGTGGCACCGAGTCGGTGCTTTTTT
GTTTTAGAGCTAGAAATAGCAAGTTAAAATAAGGCTAGTCCGTTTTTAGCGCGTGCGCCAATTCTGCAGACAAATGGCTCTAGAGAGGGC
CTATTTCCCATGATTCCTTCAT**ATTTGCAT**ATAATATTTCCTAGTAACTATAGAGGCTTAATGTGCGATAAAAGACAGATAATCTGTTCT
TTTTAATACTAGCTACATTTTACATGATAGGCTTGGATTTCTATAAGAGATACAAATACTAAATTATTATTTTAAAAAACAGCACAAAAG
GAAA**CTTACCGTAACTTGAAAGTA**ATTGTGTGTTTTGAGAC**TTTATATA**TGCATGCGAGAAAAGCCTTGTTTGGTTTGGGTCTTC

c

Oligo_hU6

5’-CTTGTGGAAAGGACGAAACACCGNNNNNNNNNNNNNNNNNNNGTTTGGGTCTTCGAGAAGACCTCACCGNNNNNNNNNNNNNNNNNNNGTTTTAGAGCTAGAAATAGC-3’

Oligo_s/mU6

5’-CATGCGAGAAAAGCCTTGTTTGGNNNNNNNNNNNNNNNNNNNGTTTGGGTCTTCGAGAAGACCTCACCGNNNNNNNNNNNNNNNNNNNGTTTTAGAGCTAGAAATAGC-3’

Supplementary Figure 2. A panel of pDonor plasmids and paired-gRNA oligos

(a) Schematic representation of pDonor plasmids. Orange box represents gRNA scaffold, green arrows represent pol III promoters. Red (mouse) and purple (human) boxes represent the various regulatory motifs present in the U6 promoters (from 5’ to 3’: the Octamer motif, the Proximal sequence element, and the TATA-box). Sizes of Donor fragments generated by BbsI digestion of each of the plasmids is shown. **(b)** Sequence present in each pDonor plasmid. BbsI sites are highlighted in brown; gRNA scaffold sequence in orange; promoter sequence in green; regulatory motifs in red (mouse) and purple (human). **(c)** Generic sequence of the oligos encoding the gRNA pairs. Homology sequence to promoter and gRNA scaffold are shown in green and orange respectively. Position of generic gRNAs is represented in red and blue.

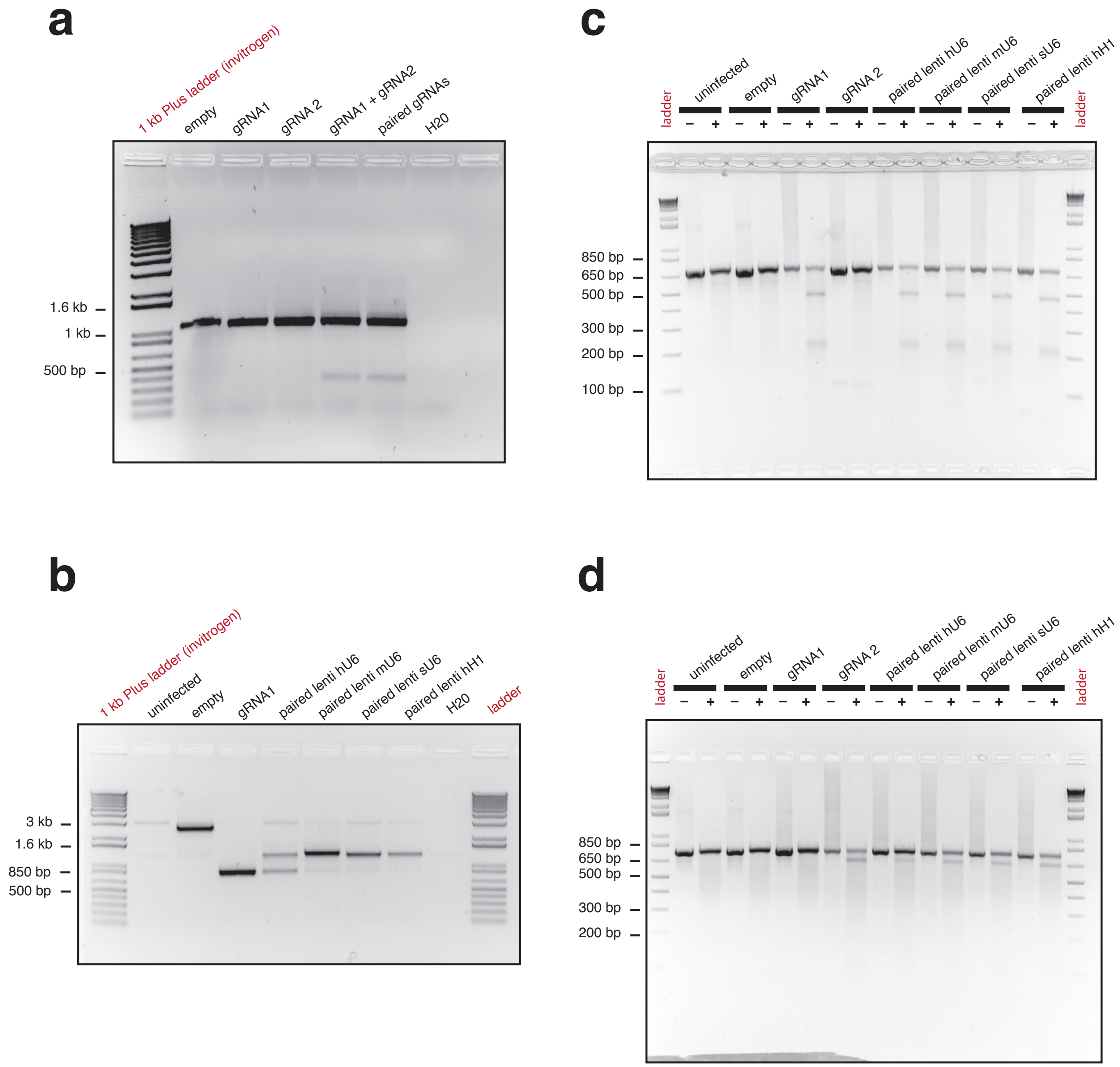

Supplementary Figure 3. Uncropped Gels

(a) PCR to detect genomic deletion (related to Figure 1d). **(b)** PCR to detect proviral recombination (related to Figure 4c). **(c)** Surveyor assay to 5’ cut site to genomic DNA of uninfected cells ot cells infected with the indicated constructs (related to Figure 4b, top). **(d)** Surveyor assay to 3’ cut site to genomic DNA of uninfected cells ot cells infected with the indicated constructs (related to Figure 4b, bottom).

Supplementary Table 1. PCR primers used in this study

Primer	Sequence
**F1**	5'- TTTCTTGGCTTTATATATCTTGTGGAAAGGACGAAAC -3'
**R1**	5'- GACTAGCCTTATTTTAACTTGCTATTTCTAGCTCTAAAAC -3'
**F2**	5'- GGCAAGTTTGTGGAATTGGT -3'
**R2**	5'- TCTCTAGGCACCGGTTCAAT -3'
**F3**	5'- AAGTTCGAGGCCATCTCTGA -3'
**R3**	5'- CAGGTGAAGTCGCTCCCTAC -3'
**F4**	5'- CCAGTACTCTCCTCCCCTCA -3'
**R4**	5'- CTCGGGTGGCTCATAAGGTA -3'
**F5**	5'- TTTGAGACTATAAATATGCATGCGAGAAAAGCCTTGTTTG -3'
**F6**	5'- TTTGAGACTTTATATATGCATGCGAGAAAAGCCTTGTTTG -3'

Supplementary Table 2. PCR amplicons and primer sets

Primer set	Purpose	Amplicon Size
**F1/R1**	Gibson Ligation of Oligo_hU6 into pDonor_hU6 fragment	148 bp
**F2/R2**	Analysis of proviral integrity	2.7 kb in empty vector; 0.8 kb in vectors with single gRNAs; 1.3 kb in vectors with paired gR-NAs
**F3/R3**	Detection of genomic deletion	~1 kb in wt locus; ~340 bp in deleted locus
**F3/R4**	5’ cut site SURVEYOR	680 bp
**F4/R3**	3’ cut site SURVEYOR	760 bp
**F5/R1**	Gibson Ligation of Oligo_s/mU6 into pDonor_mU6 fragment	148 bp
**F6/R1**	Gibson Ligation of Oligo_ s/mU6 into pDonor_sU6 fragment	148 bp

Supplementary Method: PAIRED sgRNA CLONING

We describe here a step-by-step protocol for the cloning of paired gRNA vectors. This protocol can be used with a variety of CRIRSR/Cas9 vectors, provided their linearization generates ends compatible with those in the final insert:

```
CACCGNNNNNNNNNNNNNNNNNNNNN(…425 bp…)NNNNNNNNNNNNNNNNNNNNN
    CNNNNNNNNNNNNNNNNNNNNN(…425 bp…)NNNNNNNNNNNNNNNNNNNNNCAAA
           gRNA1                              gRNA2
```

The choice of pol III promoter under which to express the distal gRNA will depend on the user and the final vector of choice. This will determine the choice of oligo, PCR amplification primers and pDonor vector. Please refer to Supplementary Tables 1 and 2 for further information.

I. INSERT PREPARATION

1. PCR amplify oligo

General PCR considerations:

- This PCR is prone to contamination, which means that the PCR mastermix should be prepared on a clean bench using clean pipettes, tips and reagents. Never bring the template DNA to this bench!

- Oligos re-suspended at 100uM have a concentration around 4ug/ul. For the amplification step I use the oligo diluted at 0.01ng/ul. Using more template gives me an unspecific band around 200bp, which I suspect is the result of autopriming between the oligos.

- For each oligo, prepare the following PCR reaction in triplicate:

Oligo (0.01ng/ul)	1 ul
10x HF Buffer	10 ul
dNTPs (10mM)	1 ul
H2O	35 ul
Forward primer (10uM)	1.25 ul
Reverse primer (10uM)	1.25 ul
Phusion polymerase	0.5 ul

- Prepare an additional reaction with water as a control
- PCR program: Anneal @ 68°C
  Extension for 30’’ @ 72°C
  25x

- Run PCR reactions on 2.5% gel and gel extract bands (~148 bp).

2. Donor fragment preparation

- Digest 10ug of pDonor_sU6 (plasmid #741) with *Bbs*I
- Gel extract 415bp band

3. Insert assembly

- Set up the following Gibson reaction for each of your oligos:

Donor fragment	405ng
Oligo amplicon	432ng
2xGibson MM	30 ul
H2O	to 60 ul

- Incubate @ 50°C for 1h

4. Nuclease Digestion **Skip this step if not cloning a pooled library**

- Add to each Gibson reaction:

10x Plasmid Safe Buffer	9 ul
ATP (25mM)	9 ul
Plasmid Safe nuclease	3 ul
H2O	9 ul

- incubate @ 37°C for 1h

5. PCR clean up and Digestion

- Clean up reactions with QIAGEN's PCR clean up kit. Elute in 50ul H20
- Digest with *Bbs*I

DNA	50ul
10x BSA (1mg/ml)	10ul
10x Buffer	10ul
*Bbs*I	3ul
H2O	27ul

- Incubate @ 37°C for 3h
- Run digestion on 2.5% gel and cut ~480 bp band
- Gel extract and elute the completed insert in 30ul. Run 5ul on a gel. You should be able to see the insert (concentration is usually around 4-5ng/ul).

II. VECTOR PREPARATION & INSERT CLONING

1. Vector preparation

- Digest 5-10ug of your vector of choice:
  - lentiCRISPR_v2 (addgene #52961; BsmBI);
  - px330 (addegen # 42230 ; BbsI);
  - …
- add 1-2ul TSAP for 1h to reduce background from partially digested molecules;
- Gel extract top band, which corresponds to vector backbone.

2. Cloning

- Set up ligations containing 50ng of vector and 6-7 ng of insert (molar ratio 1:3). Include a control ligation with water.

  NOTE: I use NEB ligase with 10x Buffer in 10ul total volume. 1h @ RT works fine. Longer incubations might increase efficiency.

- Transform bacteria with 2.5ul of ligation and select.

  NOTE: Ideally you should use recombinase deficient strains (like Stbl2) to reduce unwanted recombination between LTRs as well as between U6s. However, you need to grow these strains at 30°C which is a pain. TOP10 cells work fine for standard clonings (but beware of recombination events). Otherwise grow Stbl2 in TB (not LB) supplemented with Carbinicillin (not AMP).

- Pick colonies next day and check for positive clones by digestion (e.g. NotI/XhoI digestion for lientiCRISPR, which yields 1.8kb in positive clones. Double check if the enzymes cut within the sgRNAs). 4 colonies per cloning should be enough.

- Sequence with forward (e.g. lentiCRISPR fwd; GGCAAGTTTGTGGAATTGGT) and reverse primers (e.g. lentiCRISPR rev; TCTCTAGGCACCGGTTCAAT).
